# New daily persistent headache: a systematic review on an enigmatic disorder

**DOI:** 10.1186/s10194-019-1022-z

**Published:** 2019-07-15

**Authors:** Nooshin Yamani, Jes Olesen

**Affiliations:** 1 Headache Department, Iranian Center of Neurological Research, Neuroscience Institute, Tehran University of Medical Sciences, Tehran, Iran; 2 Danish Headache Centre and Department of Neurology, University of Copenhagen, Rigshospitalet Glostrup, Copenhagen, Denmark

**Keywords:** New daily persistent headache, NDPH, Primary headache disorders, Chronic daily headache

## Abstract

**Background:**

New daily persistent headache (NDPH) presents with a sudden onset headache which continues without remission within 24 h. Although rare, NDPH is important because it is one of the most treatment refractory primary headache disorders and can be highly disabling to the individuals*.* In this structured review, we describe the current knowledge of epidemiology, clinical features, trigger factors, pathophysiology, diagnosis and therapeutic options of NDPH to better understand this enigmatic disorder.

**Main body of the abstract:**

The prevalence of NDPH estimated to be 0.03% to 0.1% in the general population and is higher in children and adolescents than in adults. Individuals with NDPH can pinpoint the exact date their headache started. The pain is constant and lacks special characteristics but in some has migraine features. The exact pathogenic mechanism of NDPH is unknown, however pro-inflammatory cytokines and cervicogenic problems might play a role in its development. The diagnosis of NDPH is mainly clinical and based on a typical history, but proper laboratory investigation is needed to exclude secondary causes of headache. Regarding treatment strategy, controlled drug trials are absent. It is probably best to treat NDPH based upon the predominant headache phenotype. For patients who do not respond to common prophylactic drugs, ketamine infusion, onabotulinum toxin type A, intravenous (IV) lidocaine, IV methylprednisolone and nerve blockade are possible treatment options, but even aggressive treatment is usually ineffective.

**Conclusion:**

NDPH remains poorly understood but very burdensome for the individual. Multi-center randomized controlled trials are recommended to gain better understanding of NDPH and to establish evidence based treatments.

## Introduction

New daily persistent headache (NDPH) is a rare primary headache disorder, characterized by persistent headache with a particular temporal profile as it starts 1 day with a clearly remembered onset and continues in a daily pattern without remitting. NDPH predominantly affects individuals without a history of prior headache. Although the prevalence of new daily persistent headache is estimated to be rare, it is considered important because of its persistency and therapeutic refractoriness. It is very often disabling, may significantly affect the individual’s quality of life and can lead to psychiatric conditions.

NDPH as an entity has been known since 1986 when it was described by Vanast as a self-limiting and benign form of daily headache [1]. In 1988 when the first version of the International classification of headache disorder (ICHD-1) was published, NDPH was not included because of lack of data. Silberstein et al. described NDPH in 1994 as one of the chronic headache disorders in the “Silberstein-Lipton criteria” [2]. In 2004, diagnostic criteria for NDPH were included in ICHD-2 in the chapter “other primary headaches”. In the ICHD-2 diagnostic criteria, NDPH diagnosis required characteristics like chronic tension-type headache and presence of migraine features was against the diagnosis of NDPH [3]. Further observations demonstrated, however, that NDPH may sometimes have predominantly migraine features. Therefore, the diagnostic criteria in the ICHD-3β and ICHD-3 did not use any special clinical features, only sudden onset and persistence [4, 5]. Rozen has published several articles on new daily persistent headache and his review article published in 2014 discussed its definition, pathophysiology and treatment [6]. It was not a structured review and new studies have appeared since then. Therefore, a structured review is needed to increase the understanding of this enigmatic disorder.

The objective of this review is to describe the existing studies of epidemiology, clinical features, trigger factors, pathophysiology and therapeutic options of NDPH.

## Method

We performed a PubMed and EMBASE search using the terms “new daily persistent headache” and “NDPH”. In our review we restricted the inclusion criteria to papers in the English language published or e-published before February 2018. We also searched for other useful sources in the reference lists of the selected articles. After removing duplicates, there were 255 articles. One hundred forty-four were relevant to our search. After screening the title and abstract, 51 were assessed in full-text for eligibility and 40 studies were considered eligible for our structured review.

### Epidemiology

NDPH is thought to be a rare disorder, but until recently there have been limited studies of its epidemiology (Table 1). The first population-based study of NDPH was published in 1999 by Castillo et al. using the Silberstein-Lipton criteria on 1883 subjects from the general population in Spain, they found a 1-year prevalence of NDPH of 0.1% (2 cases) [7].

**Table 1:** Prevalence, age, sex and race distribution of NDPH in different studies

Reference	Location	Definition criteria	Population surveyed	NDPH prevalence	Female	Male	F:M ratio	Age of onset	Race
Castillo et. al 1999 [7]	Spain	S-L	1883 adult general population	0.1%GP					
Li 2002 [8]	USA	S-L	56 NDPH cases		40(71%)	16(29%)	2.5	12–78	Caucasian:87% Black:11% Hispanic:2%
Bigal et. al 2004 [9]	USA	S-L	170 adolescents with CDH 638 adults with CDH	21% CDH 10.8% CDH					
Takase et. al 2004 [10]	Japan	ICHD2	30 NDPH cases of 1760 CDH	1.7% CDH	13(43%)	17(57%)	0.8	13–73	
Meineri et. al 2004 [11]	Italy	ICHD2, S-L	18 NDPH cases of 265 CDH	6.7% CDH	11(61%)	7(39%)	1.6	13–76	
Mack 2004 [12]	USA	M-ICHD2	175 children with CDH	23% CDH	27(67.5%)	13(32.5%)	2.1		
Kung et. al 2008 [13]	USA	M-ICHD 2	306 children and adolescents in a tertiary headache center	28% CDH	34(64.2%)	19(35.8%)	1.7		
Grande et. al 2009 [14]	Norway	ICHD 2	30,000 adult general population	0.03% GP					
Robbins 2010 [15]	USA	M-ICHD2	71 NDPH		51(72%)	20(28%)	2.5	8–76	Cacausian:80.3% Black:5.6% Hispanic:9.9%
Prakash 2012 [16]	India	M-ICHD2	63 NDPH		36(57%)	27(43%)	1.3	18–68	
Rozen 2016 [17]	USA	ICHD-3β	97 NDPH		65(67%)	32(33%)	2	Mea: F:32.4 M:35.8	Cacausian:98% Black:1% Hispanic:1%
Uniyal et. al 2017 [18]	India	ICHD-3β	55 NDPH		45.5%	54.5%	0.8	Mea: 28.24	

*S-L* Silberstein-Lipton criteria, *ICHD* International classification of headache disorders, *M-ICHD2* Modified ICHD2 (NDPH according to the criteria A and B of the ICHD-2 regardless of the presence of migraine features.) *GP* General population, *CDH* Chronic daily headache

In a study from Norway of 30,000 persons from the general population using the more strict ICHD-II criteria, 1-year prevalence of NDPH was 0.03% in the age group 30–44 years [14]. Since the third version of ICHD has broader criteria for NDPH, the incidence of NDPH is likely to be higher.

Studies in tertiary headache centers have suggested that NDPH prevalence in children and adolescents is higher than in adults. In chronic daily headache patients, they found NDPH prevalence of 21–28% in pediatric vs 1.7–10.8% in adult patients [9, 10, 13].

NDPH may occur more in women than in men. According to some studies female to male ratio was 1.3–2.5:1, but two studies in Japan and India have shown female to male ratio of 0.8:1 [10, 16]. The age of onset varies from 8 to 78 years. Mean age of onset in adults is 32.4 years in women and 35.8 years in men [17] and 14.2 in the pediatric population [13]. The great majority of described NDPH patients (80–98%) are Caucasian [8, 15, 17].

### Clinical findings

New daily persistent headache typically presents with sudden onset headache which starts 1 day and continues without remission. Individuals with NDPH can pinpoint the exact date their headache started. Although recalling the exact date of the onset of headache was highly variable in previous studies (20–100%) [8, 11, 15, 16, 18] and a few studies even did not mention anything about it [10, 13], according to the current classification ICHD-3, distinct and clearly remembered onset is necessary for diagnosis [5]. NDPH is mostly bilateral in location and can occur anywhere in the head with mild to severe intensity (moderate intensity in most cases). The pain is constant and lacks special characteristic features but in some has characteristics of migraine (including unilateral pain, pulsating quality, worsening by physical activity, photophobia, phonophobia, nausea and vomiting) [8, 15].

NDPH typically develops in individuals with no or insignificant previous headache history. However, patients with prior episodic headache are not excluded from NDPH diagnosis if NDPH is different from the previous headache and they do not describe increasing headache frequency prior to the its onset or association with medication overuse [5].

Although about 30–50% of patients in different case series reported a family history of unspecified headache, none of them mentioned occurrence of the same disorder in other family members [8, 15].

Comorbid symptoms in NDPH patients include sleep disturbances, light-headedness, blurred vision, neck stiffness, concentration problems, sensory disturbances such as numbness or tingling, vertigo, lethargy and other non-specific syndromes [8]. Mood disorders are considerably more prevalent in NDPH in comparison to healthy subjects. In a study of psychiatric comorbidity among NDPH patients, severe anxiety was seen in 65.5% and severe depressive symptoms in 40% [18]. Clinical features of the NDPH patients from different studies are detailed in Table 2.

**Table 2:**
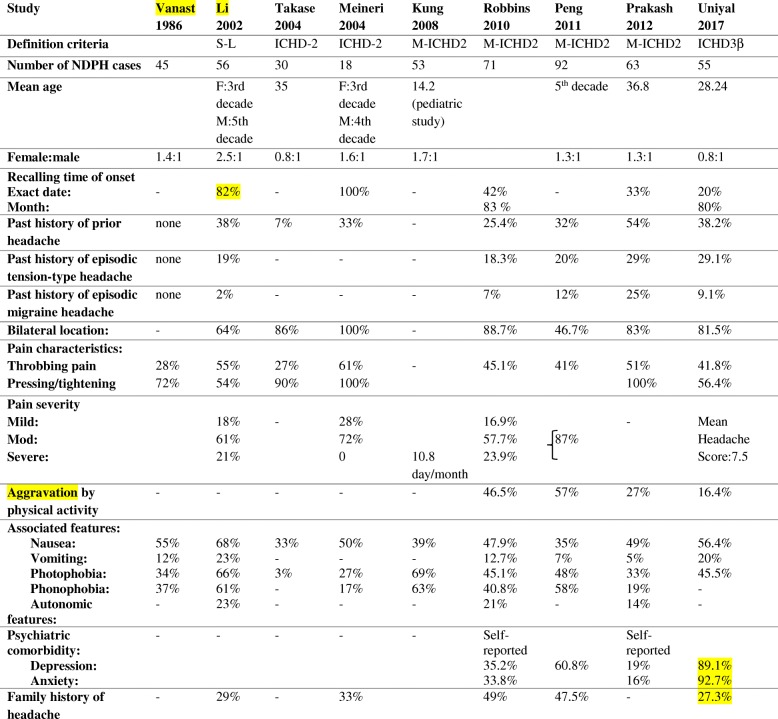
Clinical characteristics of patients with NDPH in various published studies

^a^[1]

^b^[8]

^c^[10]

^d^[11]

^e^[13]

^f^[15]

^g^[20]

^h^[16]

^i^[19]

### Precipitating factors

Multiple prior studies demonstrated that a number of factors might precipitate NDPH. Recognizing the precipitating events might help to understand NDPH pathogenesis. Rozen in 2016 looked at precipitating events in 97 NDPH patients in a headache specialty clinic population. For both males and females, the majority (53%) could not recognize a precipitating factor. Precipitating events were noted in 47% of patients with an infection and flu-like illness being the most common (22%), while stressful life events were noted in 9% of patients. In 9% NDPH was triggered by surgical procedures with intubation while 7% had some “other” recognized trigger [17] (Table 3).

**Table 3:** Patient reported NDPH triggers in various published studies

Reference	Number of NDPH patients	No Triggering factor	Infection or flu-like illness	Stressful life event	Trauma /surgery	Other
Li 2002 [8]	56	> 33%	30%	12%	12%	
Mack 2004 [12]	40 (pediatric NDPH)	5(12%)	17(43%)		13(33%)	5(12%)idiopathic intracranial hypertension, high altitude climbing
Takase 2004 [10]	30	24(80%)	^a^	6(20%)	^a^	
Robbins 2010 [15]	71	38(53.5%)	10(14.1%)	7(9.9%)		6(8%)menarche, SSRI withdrawal, HPV vaccination
Peng et. al 2011 [19]	92	65(71%)	3(3%)	24(26%)		
Prakash 2012 [16]	63	29(46%)	18(29%)	5(8%)	10(16%)	9(14%) postpartum, medication overuse
Rozen 2016 [17]	97 Female:65 Male:34	51(53%) Female: 52% Male: 53% Mean age:30.4	21(22%) Female: 22% Male: 22% Mean age:31.8	9(9%) Female: 11% Male: 6% Mean age:28.1	9(9%) Female:9% Male:9% Mean age:63.3	7(7%) syncope, hormone, toxin and medication, cervical massage
Uniyal et. al 2017 [18]	55	35(63.5%)	10(18%)	5(9.1%)	5(9.1%)	

^a^Takase et al. excluded persistent headache occurred in relation to an infection or flu-like illness and headache after head and neck injury or surgery

There was no significant difference between males and females in precipitating events or for frequency or occurrence of any of the precipitating factors. Mean age of onset was significantly higher in the post-surgical subgroup (63.3 years) than in post stressful life event (28.1), no precipitating event (30.4) and post infection (31.8). No significant difference was reported between patients who had a history of migraine vs no migraine and aside from stressful life event, existence of prior migraine headache did not increase the frequency of precipitated vs non-precipitated NDPH [17].

In a study of 40 pediatric headache patients with NDPH, precipitating events were noted in 88%: febrile illness in 43%, preceding minor head injury in 23% and cranial or extra cranial surgery in 10% [12].

In most subsequent studies, infection, stressful-life event and extracranial surgical procedure have been described to precipitate NDPH. Other reported precipitating factors include withdrawal from SSRIs, human papilloma virus vaccination, menarche and postpartum state, hormone manipulation with progesterone, toxin and medication exposure, cervical massage treatment, simple syncopal attack and thyroid diseases [12, 15–17]. None of these studies discuss whether in the presence of a precipitating event, the diagnosis of NDPH can be maintained. If head trauma or infection precipitates, it would be more appropriate to have the diagnosis of “headache attributed to injury to the head” or “headache attributed to infection”.

### Pathogenesis

Unfortunately, very few have studied the pathogenesis of NDPH and we still know very little about it. A significant portion of NDPH patients describe that they experienced infection or a flu-like illness at the onset of headache. Some authors have associated NDPH to Epstein-Barr virus (EBV) infection. In a case-control study, Diaz-Mitoma demonstrated that 84% (27) of 32 NDPH patients had evidence of active EBV infection compared to 25% in a gender and age matched control group [20]. In another study 23% (9) of 40 children with NDPH had positive EBV serology [12]. Li and Rozen tested EBV titers in seven NDPH patients of their series and they noticed five out of seven patients had positive titers against EBV suggestive of former EBV infection [8]. Meineri et al. in a case series of 18 NDPH patients did not identify any EBV infection, but they found evidence of recent Herpes simplex virus (HSV) infection in 42% (6 patients) and of Cytomegalovirus (CMV) in 11% (2 patients) [11]. Other associations have been made with Herpes zoster, Adenovirus, Toxoplasmosis, Salmonella, Streptococcal infection and *Escherichia coli* urinary tract infections [21].

Considering that a certain percentage of patients appear to develop NDPH after an infection, Rozen and Swidan proposed that NDPH might develop in response to the release of pro-inflammatory cytokines during persistent systemic or CNS inflammation and looked at Tumor necrosis factor alpha (TNF-α) levels in the cerebrospinal fluid (CSF) and serum of NDPH patients to discover whether increased level of pro-inflammatory cytokines due to CNS inflammation might lead to NDPH evolution. In 19 out of 20 NDPH patients from an inpatient headache unit, TNF-α levels were high in CSF samples. However, serum TNF-α levels were normal in most patients. The authors then suggested that in NDPH, pain might be due to chronic central nervous system inflammation, cytokine production and persistent glial activation that arise in response to precipitating events [22].

Rozen et al. noticed that their NDPH patients had characteristics similar to patients with connective tissue disorders. They were thin, tall, had a long neck and on physical examination they had lax joints suggestive of underlying cervical spine and systemic joint hypermobility. Using Beightons score as a screening test for joint hypermobility in 12 NDPH patients, they revealed that 11 had cervical spine joint hypermobility and 10 had widespread joint hypermobility. Thus, they suggested a possible role for cervical spine joint hypermobility in the pathogenesis of NDPH [23].

In another study, all 9 post-surgical NDPH cases in Rozen’s material had endotracheal intubation. Thus, he suggested a cervicogenic origin to their headache caused by the cervical hyperextension during neck positioning for intubation [17].

On balance it seems that most proposed pathogenic mechanisms are somewhat speculative. Infections are enormously prevalent in the general population and it is only a tiny number who get NDPH after infections. The proposed intrathecal inflammation was not a controlled study and there has been no other indication of inflammation in these patients. Mild head trauma cannot be counted as a cause of NDPH because it has to be diagnosed as headache attributed to injury to the head. Thus, NDPH remains enigmatic and in need of further controlled studies of its mechanism.

### Diagnosis of NDPH

The diagnosis of NDPH is based on a typical history and usually the neurological and general examination and neuroimaging studies are unremarkable. Rozen retrospectively studied brain MRI findings of 97 primary NDPH patients. According to this study, white matter abnormalities or infarct-like lesions do not appear to occur in this condition, unless there is accompanying cardiovascular or cerebrovascular risk factors. Nevertheless, neuroimaging study is necessary to exclude several brain disorders particularly spontaneous CSF leak and cerebral venous sinus thrombosis that can mimic NDPH (Table 4). A gadolinium-enhanced brain MRI with MR venography is recommended in all patients. If there is any doubt about the presence of aneurysms or arterial dissections, then intracranial and extracranial MR or CT angiography is warranted [6, 24]. A lumbar puncture with CSF manometry may be indicated, especially in treatment refractory cases. According to European Headache Federation consensus on investigation for primary headache disorders, viral titers for Epstein Barr virus can be beneficial in selected patients. However, Rozen suggested that all patients with NDPH should have viral titers drawn (IgG, IgM) for Epstein Barr virus, cytomegalovirus, human herpes virus type 6, and parvovirus [6].

**Table 4:** Secondary mimics of NDPH

• Low or raised CSF pressure (Spontaneous CSF leak, Idiopathic intracranial hypertension, Intracranial mass lesion) • Cerebral venous thrombosis • Cranial artery dissection • Cranial arteritis • Posttraumatic headache (subarachnoid hemorrhage, subdural hematoma, …) • Meningitis • Sphenoid sinusitis • Contact-point headache (caused by contact of intranasal structures)	

### Treatment

NDPH is known as one of the most treatment refractory primary headache types. There have been only a few studies reviewing NDPH treatment up to now and there is no specific well-defined strategy for its treatment in the absence of double-blind controlled studies. In clinical practice, most headache specialists treat NDPH based upon the prominent headache phenotype, whether migrainous or tension type. However even aggressive treatments are usually ineffective or only partially effective. NDPH patients are therefore prone to overuse medications. A few treatment regimens for NDPH have been studied in the literature:

#### Methylprednisolone

In one study, Prakash and Shah observed treatment response to a course of 5-days high dose methylprednisolone in 9 post-infectious NDPH patients. Six of them also received oral steroids for 2–3 weeks following intravenous methylprednisolone. All patients reported improvement. Seven had almost full recovery within 2 weeks, while in two other patients complete pain relief occurred within 1.5 to 2 months after starting the treatment [25]. The weakness of this study is that 5 of 9 patients were treated just few weeks after the headache began while the ICHD diagnostic criteria required at least 3 months of headache for NDPH diagnosis. Thus, treatment with high dose IV corticosteroids may not be as favorable in some classic cases that fulfill ICHD-3 diagnostic criteria.

#### Tetracycline derivatives

Doxycycline is a drug recognized to inhibit TNF-α. In a small, open-label trial reported in an abstract by Rozen [26], four treatment refractory NDPH patients with high TNF-α levels in the CSF were given 100 mg doxycycline twice daily for 3 months. Three patients reported that their headache had been precipitated by an infection. All patients had improvement within 3 months of initiation of doxycycline. Complete relief of the pain occurred in two NDPH patients who had the highest CSF TNF-α levels, while one patients reported 80% decrease in pain intensity, and one experience more than 50% decrease in frequency of severe headache episodes with minor reduction in severity of daily headaches.

Rozen, has described some effects for montelukast (10 mg twice daily) when added to doxycycline or minocycline to treat NDPH. However, there is no evidence in the literature to support using montelukast in the treatment of NDPH [6].

#### Topiramate and gabapentine

Rozen presented 5 NDPH patients in an abstract with favorable response to either gabapentin or topiramate but again no good scientific evidence supports using these medications for treatment of NDPH [6].

#### Mexiletine

Marmura et al. in a retrospective study reported on patients with refractory chronic daily headache including 3 NDPH patients who had been treated with mexiletine. All 3 NDPH cases reported decrease in pain intensity, while only one had diminished headache frequency. Serious adverse effects were reported during the treatment [27].

#### Nerve blockade

Robbins et al. performed nerve blocks in painful areas with 0.5% bupivacaine in 23 NDPH patients. It provided 60% acute response, consistent with at least one-day decrease in pain intensity in patients with NDPH [15].

In a retrospective review, Hascalovici et al. reported treatment response of 67% with peripheral nerve blockade in 3 NDPH patients. They considered nerve blockade as a safe and efficient strategy to treat older NDPH patients [28].

Puledda et al. reported that improvement was seen in 13 of 22 (59%) children and adolescents with NDPH who received greater occipital nerve block using 1% lidocaine and methylprednisolone [29].

#### Onabotulinum toxin type a (BTX)

In a case report, Spears treated a 67-years-old NDPH patient with 3 rounds of BTX injection. He reported 8–12 weeks of absolute pain free periods after each treatment [30].

Trucco and Ruiz reported a 19-year-old woman with refractory NDPH who had partial relief after the first injection of BTX and almost complete response after the third cycle [31].

Tsakadze and Wilson reported pain relief of 75% in one and 100% in one patient with treatment refractory NDPH who were treated with BTX injection every 3 month [32].

#### Intravenous lidocaine

Marmura et al. in a retrospective study, studied 68 intractable cases with chronic daily headache including 12 NDPH patients were treated with IV lidocaine. 25.4% of subjects exhibited a complete response and 57.1% exhibited partial response. They suggested that patients with NDPH may benefit from IV lidocaine treatment [33].

Akbar reported a 16-year-old boy diagnosed as NDPH who was refractory to several aggressive inpatient therapies. He was treated with IV lidocaine infusion and reported that the headache fully resolved for 2 weeks and severity and frequency decreased for almost 3 months [34].

#### Intravenous dihydroergotamine (IV DHE)

Nagy et al. studied the effect of IV DHE in the treatment of refractory primary headache disorders. Two of 11 NDPH cases in their study reported only mild benefit from DHE therapy. Both had migranous features. Thus, they proposed that in contrast to the effect of IV DHE in the chronic migraine, the outcome for treatment of NDPH with IV DHE particularly those with non-migranous characteristics is less encouraging [35].

#### Intravenous ketamine

In a retrospective study, Pomeroy et al. treated 14 NDPH patients who had previously failed aggressive treatments with a sub-anesthetic dose ketamine infusion. Acute response was seen in 8 (57.1%) NDPH patients receiving ketamine, while half of them reported persistent effect of it. As it is well tolerated, a trial of ketamine might be considered reasonable in refractory NDPH cases [36].

#### Osteopathic manipulation treatment

Alexander reported a 15-year-old girl with NDPH who had pain relief after osteopathic manipulation treatment. He proposed that osteopathic manipulation treatment might be helpful in treatment resistant NDPH cases [37].

#### Nimodipin

Rozen et al. presented a 46-year-old woman with NDPH started as thunderclap headache followed by 13 month of daily headache from onset along with acalculia. All symptoms resolved rapidly and completely with nimodipin 30 mg administered twice daily. He proposed this case as a distinct subtype of NDPH caused by continuous cerebral artery vasospasm due to rapid increase in CSF TNF-α levels. This is the only report of efficacy of nimodipin in NDPH [38].

#### Combination of various drugs

Prakash et al. treated 37 NDPH patients with a combination therapy of IV methylprednisolone, IV sodium valproate, anti-depressant (amitriptyline or dothiepin) and naproxene for at least 3–6 months. After a median follow-up of 9 months, the clinical response was “excellent” (no or less than 1 headache per month) in 37% and “good” (50% reduction in headache frequency or days per month) in 30% of NDPH patients [16].

In summary, ketamine infusion, onabotulinum toxin type A, intravenous (IV) lidocaine, IV methylprednisolone and nerve blockade are possible treatment options for patients who do not respond to common prophylactic drugs.

A few reports have suggested a better response when adequate treatment of NDPH administered early in the course of the disease (within 3–12 months of NDPH onset) [16, 39]. However, this association has not been established in all studies [10].

### Prognosis

According to the ICHD-3 classification, NDPH has two sub-types: a self-limiting form, which typically resolves within a few months and a refractory form, which is resistant to aggressive treatment [5].

NDPH prognosis was initially thought to be benign. In the original report of NDPH, Vanast found that 78% of NDPH patients were pain-free without treatment within 24 months [1]. In a later series of 18 NDPH patients, 66% were headache-free by 24 months [11]. However, in subsequent studies and in clinical practice, NDPH is more likely to persist for many years and be refractory to treatment. In a study of 56 NDPH patients by Li and Rozen, the duration of headache at study entry was at least 6 months in all patients. Many patients in their series had NDPH for more than 5 years and in a few, headaches lasted for more than 10 years [8]. In a series of 30 NDPH patients from Japan, the mean duration of headache at study entry was 3.3 years, ranging from 3 months to 27 years [10]. Robbins et al. in a retrospective chart review, studied the clinical and prognostic course of 71 NDPH patients. In 76%, headache was continuous without remission from the onset grouped as persisting subform. The median duration of headache was longer in persisting NDPH patients with migraine features (31 month) than those who had characteristics like tension-type headache (18 months). In 15.5%, patients described complete or partial remission with headache occurring no more than 4 days per month for at least 3 months (remitting subform) and 8.5% in their series experienced persistent headache associated with remission periods (relapsing-remitting subform). The median duration of the remitting subform was 21 months and in the relapsing-remitting subgroup the median duration before the first remission was 5.5 months. They combined the remitting and the relapsing-remitting subforms and suggested further classifying NDPH patients into two prognostic subforms: persisting subform and nonpersisting subform. Patients in the persisting subgroup were more likely to be of white race and having history of anxiety or depression. The median age of onset was older for men in the persisting subform (28 vs 16 years), and for women in the nonpersisting subform (34 vs 24 years). No significant difference was noted among the prognostic subforms in most aspects including headache features, triggering events, history of prior headache, family history, onset and treatment aspects [15]. According to the literature it is not possible to differentiate both subtypes clinically and it is unclear whether there is any time line to differentiate self-limiting to refractory subtype. In Robbin’s series, over half of the NDPH patients with persisting subform experienced continuous daily headache for 24 months or longer. Among patients with remitting subform, remission occurred within 24 months in 63.3% and all patients in the relapsing-remitting subgroup, remitted for the first time within 24 months [15]. Long-term prognosis of persisting NDPH is still unknown.

## Conclusion

NDPH remains poorly understood but very burdensome for the individual. Multi-center randomized controlled trials are recommended to gain better understanding of NDPH and to establish evidence based treatments.

## Data Availability

The datasets used and/or analysed during the current study are available from the corresponding author on reasonable request.

## Abbreviations

BTX: Botulinum toxin
CMV: Cytomegalovirus
CSF: Cerebrospinal fluid
DHE: Dihydroergotamine
EBV: Epstein-Barr virus
HSV: Herpes simplex virus
ICHD: International classification of headache disorder
IV: Intravenous
NDPH: New daily persistent headache
TNF-α: Tumor necrosis factor alpha
